# The Molecular Basis for E^rns^ Dimerization in Classical Swine Fever Virus

**DOI:** 10.3390/v13112204

**Published:** 2021-11-02

**Authors:** Manjula Mischler, Gregor Meyers

**Affiliations:** Institut für Immunologie, Friedrich-Loeffler-Institut, Südufer 10, Insel Riems, 17493 Greifswald, Germany; manjula.mischler@gmx.de

**Keywords:** pestivirus, flavivirus, envelope protein, dimerization of glycoprotein, amphipathic helix, disulfide bond formation, virulence factor, RNA virus

## Abstract

The pestivirus classical swine fever virus (CSFV) represents one of the most important pathogens of swine. Its virulence is dependent on the RNase activity of the essential structural glycoprotein E^rns^ that uses an amphipathic helix as a membrane anchor and forms homodimers via disulfide bonds employing cysteine 171. Dimerization is not necessary for CSFV viability but for its virulence. Mutant E^rns^ proteins lacking cysteine 171 are still able to interact transiently as shown in crosslink experiments. Deletion analysis did not reveal the presence of a primary sequence-defined contact surface essential for dimerization, but indicated a general importance of an intact ectodomain for efficient establishment of dimers. Pseudoreverted viruses reisolated in earlier experiments from pigs with mutations Cys171Ser/Ser209Cys exhibited partially restored virulence and restoration of the ability to form E^rns^ homodimers. Dimer formation was also observed for experimentally mutated proteins, in which other amino acids at different positions of the membrane anchor region of E^rns^ were replaced by cysteine. However, with one exception of two very closely located residues, the formation of disulfide-linked dimers was only observed for cysteine residues located at the same position of the helix.

## 1. Introduction

Members of the genus *Pestivirus* in the family *Flaviviridae* belong to the most economically important viruses of livestock. The family also comprises the genera *Flavivirus*, *Hepacivirus* and *Pegivirus* [1,2]. The genus *Pestivirus* originally encompassed only four species, two types of bovine viral diarrhea virus (BVDV-1 and BVDV-2), classical swine fever virus (CSFV) and Border disease virus (BDV) of sheep. Recently, a significant number of further genus members were identified in pigs and a variety of new host species [3,4]. Pestiviruses have single-stranded, positive-sense RNA genomes of ~12.3 kb length that contain one long open reading frame coding for a polyprotein of about 4,000 amino acids. Translation of the genomic RNA leads to a hypothetical polyprotein, which is co- and post-translationally processed into at least 12 mature proteins [5,6]. Four viral proteins—C, E^rns^, E1 and E2—are found in the virion [7,8]. Except for the capsid protein C [9], all of these proteins are essential for recovery of infectious virus particles [10,11,12,13]. Pestiviruses exhibit significant similarity with human hepatitis C virus (HCV) with regard to the basic molecular features [5]. The most obvious difference between the members of the two genera at the genome level is the presence of two additional protein-coding regions in the pestivirus ORF [6]. These pestivirus-specific sequences code for the non-structural protein N^pro^ and the viral envelope protein E^rns^. N^pro^ is encoded by the 5′ terminal part of the pestivirus ORF. It has autoprotease activity which is only necessary for its release from the nascent protein chain [14,15]. N^pro^ is not essential for virus replication in tissue culture cells [16,17,18], but blocks the host cellular type 1 and type 3 interferon (IFN) response to virus infection and to the presence of double stranded RNA in the cytoplasm [17,18,19,20,21,22].

The E^rns^ protein is an essential structural protein necessary for recovery of infectious pestiviruses. Deletion of the E^rns^-coding region from the viral genome resulted in replicons capable of autonomous RNA replication but unable to produce infectious virus particles [11,13]. However, E^rns^ is also important for blocking the host response to pestivirus infection. The activity of E^rns^ with regard to blocking the host response is connected with its unique intrinsic RNase activity and involved in the establishment of persistent infections as best studied for BVDV [18,23,24,25,26]. E^rns^ accomplishes its association with the viral envelope via an amphipathic helix located in its utmost carboxy-terminal region [11,27,28,29,30]. This unusual membrane anchor is crucial for processing of the E^rns^-E1 precursor, for intracellular retention of the mature E^rns^ in the ER and for secretion of considerable amounts of the protein into the extracellular space [7,8,10,24,31,32,33,34]. E^rns^ is important for the virulence of pestiviruses since several recombinant pestiviruses with mutated E^rns^ coding sequences are clinically attenuated [25,35,36].

Monomeric E^rns^ protein has a size of ca. 45 kDa, nearly half of which is due to glycosylation [7,34,37]. It contains eight cysteines that form intramolecular disulfide bonds and are conserved in all pestiviruses analyzed so far [38,39]. A ninth cysteine residue is found rather close to the carboxy-terminal end of the protein in the overwhelming majority of pestivirus isolates [40]. This cysteine represents residue #171 of the E^rns^ protein (C438 or C441 in the polyproteins of CSFV or BVDV, respectively) and is engaged in the formation of E^rns^ dimers via disulfide bonds between two monomers of the protein [7,37,40]. These homodimers are found both in infected cells and the virus particle, but are not necessary for pestivirus viability [7,40]. In earlier work we have shown that CSFV mutants unable to establish stable E^rns^ dimers were attenuated in their natural host. This finding pointed at a connection between the ability to express E^rns^ homodimers and virulence. This conclusion was strongly supported by the surprising isolation of pseudorevertants from infected animals still lacking C171 but containing a mutation S209C in E^rns^. These pseudorevertants regained the ability to generate E^rns^ homodimers and displayed partially restored virulence in the natural host [41]. The so far available data leave some questions open. One question concerns the role of position 209: is this position especially suited for pseudoreversion, or can Cys residues at other positions also be used for dimerization? It was also not clear whether E^rns^ homodimers are in general dispensable for pestivirus propagation or whether noncovalently linked dimers are still present in the absence of C171. Such structures could be formed via non-covalent protein/protein interaction and be only short-lived to allow establishment of the disulfide bond formation, or could be considerably stable as was shown for the HCV E1 homotrimers and E1/E2 heterodimers [42,43,44,45,46,47].

## 2. Materials and Methods

### 2.1. Cells and Viruses

SK6 cells were obtained from A. Summerfield (Institut für Virologie und Immunologie, Mittelhäusern, Switzerland). BHK-21 cells were kindly provided by T. Rümenapf (Veterinärmedizinische Universität, Vienna, Austria). All cells were grown in Dulbecco’s modified Eagle’s medium supplemented with 10% fetal calf serum and nonessential amino acids. The fetal calf serum was tested for absence of BVDV and anti BVDV antibodies. Vaccinia virus MVA-T7 [48] was kindly provided by G. Sutter (Ludwig Maximilian University Munich, Munich, Germany).

### 2.2. Construction of Plasmids

Restriction and subcloning were done according to standard procedures [49]. Unless stated otherwise, all restriction and modifying enzymes were purchased from New England Biolabs (Frankfurt am Main, Germany) and Thermo Fisher (Karlsruhe, Germany). Synthetic DNA oligonucleotides were obtained from Metabion (München, Germany).

The plasmid SE^rns^ containing the E^rns^ coding sequences of CSFV Alfort/Tübingen (originally named SSeqE^rns^ [32,34]) was used as the basis for the mutant expression constructs tested here. Single and multiple point mutations were introduced with standard PCR based methods with thermostable Pfu polymerase (Promega, Heidelberg, Germany) and synthetic primers (QuickChange mutagenesis) in one single reaction or consecutive approaches. Establishment of the deletion library was described before; additional deletion constructs used in the present work were established the same way as described [50]. Similarly, the establishment of constructs coding for E^rns^ proteins with carboxy-terminal V5-tag extensions were done as described before for BVDV E^rns^ [40]. The mutated PCR products and plasmid constructs were all verified by nucleotide sequencing with the BigDye Terminator Cycle Sequencing Kit using a 3130 Genetic Analyzer (both PE Applied Biosystems, Weiterstadt, Germany). Sequence analysis and alignments were done with Geneious PrimeR software (Geneious Prime 2019.2.3) (Biomatters, Ltd. Auckland, New Zealand). Further details of the cloning procedures and the sequences of the primers used for cloning and mutagenesis are available on request.

### 2.3. Expression, Metabolic Labeling, and Immunoprecipitation of Proteins

BHK-21 cells were infected with vaccinia virus MVA-T7, subsequently transfected with the desired cDNA construct using SuperFect (Qiagen, Hilden, Germany) and labeled with Tran35S-Label (ICN-MP Biochemicals, Eschwege, Germany or Hartmann Analytic, Göttingen, Germany) as described earlier [40]. Supernatant of the cell cultures was harvested for determination of secreted proteins; the cells were washed twice with PBS before cell extracts were prepared under denaturing conditions. Protein expression in equivalent amounts of cell-free supernatant and cell extract was analyzed via immunoprecipitation as described before [10] using monoclonal antibody 24/16 [37] or a polyclonal rabbit serum against the carboxy-terminal sequence of E^rns^ for detection of E^rns^. Precipitates were treated before electrophoresis with 1 µL PNGase F (New England Biolabs, Frankfurt am Main, Germany) for 1 h at 37 °C, as suggested by the supplier.

### 2.4. Gel Electrophoresis, Detection, and Quantification of Precipitated Proteins

The precipitated proteins were separated by 10% SDS-PAGE (gel system as published [51]) and E^rns^ detected and quantified with a Fujifilm BAS-1500 or a CR-35 Bio image plate scanner (Elysia-Raytest, Straubenhardt, Germany). The intensities of the signals were determined with TINA 2.0 or AIDA Image Analyser 5 (both software programs from Elysia-Raytest). For quantification of E^rns^ dimers, the E^rns^ monomer signals were added to those determined for the dimer signals to obtain 100% expression product as a basis for calculation of the percentage of homodimer. The presented data represent the averages of at least three independent experiments. Statistical analysis in the form of a two-tailed t test was performed using the GraphPad Prism software (STATCON GmbH, Witzenhausen, Germany).

### 2.5. DSP Crosslink

To detect proteins that interact only transiently and without formation of covalently linked or highly stable noncovalent oligomers, we used an in vivo crosslink approach. Briefly, cells in a 3.5 cm culture dish transiently expressing the desired proteins were in vivo labelled as described in 2.3, washed two times with PBS, followed by the addition of 1 mL of 1mM DSP [dithiobis(succinimidyl propionate)] solution (generated by mixing 40 µL of 25 mM DSP in DMSO with 960 µL of PBS) and incubation for 30 min in a cell culture incubator. Thereafter, 20 µL of 1M Tris pH 7.5 was added to stop the DSP reaction, followed by washing the cells with PBS. Lysis and immunoprecipitation were done as reported before [40]. DSP was purchased from Thermo Fisher.

### 2.6. Establishment of Helical Wheel Representation

The original wheel representation was established by using the software http://rzlab.ucr.edu/scripts/wheel/wheel.cgi (accessed April 2015). The resulting scheme was subsequently edited by hand to highlight important residues etc.

## 3. Results

### 3.1. E^rns^ without Cysteine 171 Forms Transient Dimers

**Figure 1:**
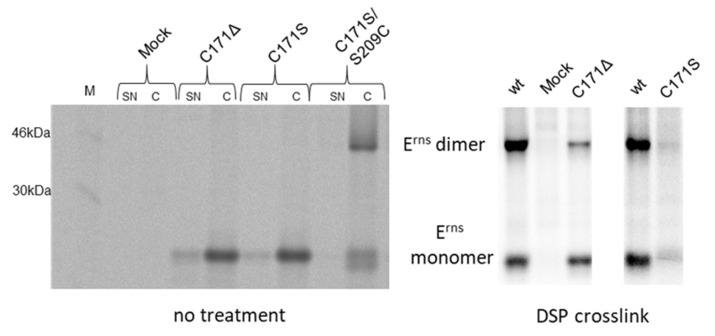
Monomers of E^rns^ lacking C171 form dimers when subjected to DSP crosslink. Results of immunoprecipitation experiments of proteins transiently expressed from constructs coding for E^rns^ wt or mutants with the indicated alterations. Proteins were radioactively labelled in situ and either left untreated (**left** part) or subjected to DSP crosslink prior to cell lysis (**right** part). Precipitation was done with E^rns^-specific monoclonal antibody 24/16 [37]. Precipitates were treated with PNGase F prior to separation by 10% SDS-PAGE under nonreducing conditions. M: bands of a size marker. Mock cells were infected with MVA-T7 and incubated with radioactive amino acids but not transfected with a plasmid. For the experiment shown on the left, both cell extract (C) and cell culture supernatant (SN) were analyzed. Since the supernatant did not contribute to the subject of the experiment, we omitted SN analysis when conducting the crosslink experiment. As obvious, the right part represents lanes from two different gels. Therefore, the wt control is shown twice here.

We have shown before that cysteine 171 in E^rns^ is engaged in homodimerization. Upon deletion or exchange of C171, dimers could no longer be detected via nonreducing SDS-PAGE or blue native gel electrophoresis (BNE) [40] that would allow detection of stable but noncovalently linked dimers. Accordingly, homodimers of pestiviruses cannot be detected in the absence of C171. However, one can speculate that E^rns^ monomers must transiently interact to allow the efficient formation of the disulfide bond. We therefore conducted a crosslink experiment with DSP that was added to the medium of cells transiently expressing CSFV E^rns^ or an E^rns^ mutant with a deletion of C171. A control in the absence of a crosslinker showed the expected pattern after immunoprecipitation with a monomer and dimer for the wt protein, but only a monomer for the mutant (Figure 1). In the presence of a crosslinker, both proteins yielded homodimers that were resolved into monomers under reducing conditions (Figure 1). Interestingly, no smear pointing at the formation of E^rns^ crosslinked to various cellular protein partners was observed, a result which strongly supports the hypothesis of a specific interaction of two E^rns^ monomers and not a crosslink of molecules that were accidentally located in close vicinity.

### 3.2. E^rns^ Does Not Contain a Specific Localized Contact Domain for Transient Interaction of Monomers

**Figure 2:**
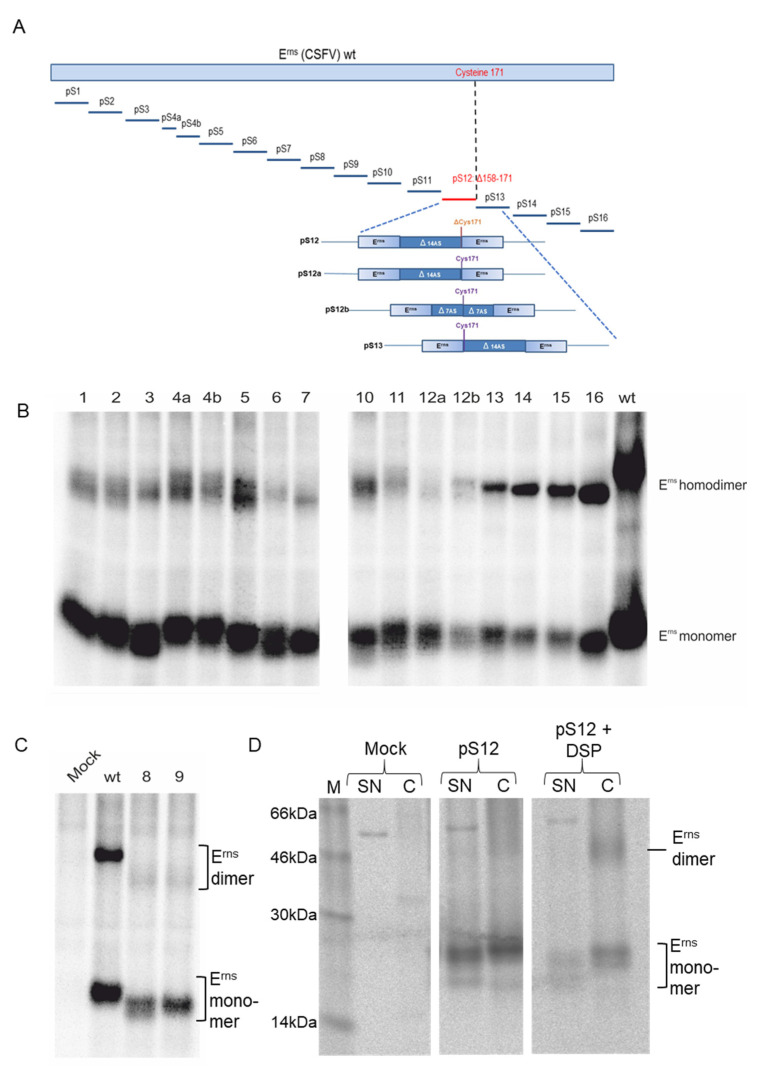
Influence of deletions of E^rns^ sequences on homodimer formation. (**A**) Schematic representation of the E^rns^ protein (upper part) and the location of the deletions introduced into the constructs. In most cases, the deletions encompassed 14 amino acids, but due to instability, deletion mutant #4 had to be divided into two smaller sections. In addition, the region around Cys171 was analyzed with four different constructs, either deleting the Cys codon (pS12) or preserving it at the 3′ or 5′ terminal end of the deletion (pS12a or pS13, respectively). The fourth construct pS12b encoded a protein with two deletions of seven codons flanking codon 171. (**B**) Results of an immunoprecipitation experiment of proteins transiently expressed from the constructs shown in (**A**). Precipitation was done with E^rns^-specific monoclonal antibody 24/16 [37]. Precipitates were treated with PNGase F prior to separation by SDS-PAGE under nonreducing conditions. (**C**) Equivalent to (**B**), but since constructs pS8 and pS9 lack the epitope recognized by mab 24/16, precipitation was done with the polyclonal rabbit serum K81 directed against the carboxyterminal region of E^rns^. (**D**) Precipitated proteins expressed from construct pS12 without or with DSP crosslink (middle or right part, respectively). In contrast to the constructs analyzed in (**B**), the deletion in pS12 includes the Cys at position 171 [see also (**A**)]. We analyzed cell lysate (**C**) and supernatant (SN) here.

The crosslink experiment showed that two E^rns^ monomers associate at least transiently and form homodimers too unstable to be detected via BNE. This finding raises the question whether a specific interaction domain exists that is necessary for initiating stable homodimerization by aligning monomers and giving time for disulfide bond formation. To test whether such a locally restricted interaction surface exists, we analyzed homodimerization of a set of deletion mutants. The respective expression plasmid library was already described and used before [50]. Briefly, the plasmids code for E^rns^ proteins with consecutive deletions of 14 amino acids. We chose this size because we expected that the loss of 14 residues should be sufficient to impair motifs based on primary structure significantly without inducing a strong tendency towards complete destruction of the structure, that would make dimer formation highly unlikely or even make the protein unstable. In one case, a 14mer deletion resulted in an unstable product, so we had to split this peptide and establish two constructs with smaller deletions of five and nine amino acids (Figure 2A). Transient expression with subsequent immunoprecipitation and SDS-PAGE under non-reducing conditions showed that all deletion mutants were able to form disulfide-linked homodimers, though with obviously reduced dimer to monomer ratios for some mutants, especially constructs 6, 7, 8, 9 and 11 (Figure 2B,C). The only construct that did not yield a homodimer band was pS12, in which the deletion encompasses the codon C171, so that formation of S-S bridged (disulfide bridged) dimers is not possible (Figure 2D). We therefore established two further constructs preserving the cysteine residue but deleting either the last fourteen residues upstream of C171, or the seven residues upstream and the seven residues downstream of this residue (constructs pS12a and pS12b, respectively). For both constructs homodimerization could be detected, but the homodimer bands were again quite weak, especially for p12a. We therefore conducted a further crosslink experiment with construct pS12 and detected a homodimer in the cell extract that was not present without the crosslink (Figure 2D). We also tested the cell culture supernatant here, but signals clearly supporting the generation of covalently linked dimers were not obtained. Taken together, the sequence surrounding C171 might have an influence on the efficiency of disulfide bond formation, but is not crucial for initiation of homodimerization.

In general, the amounts of homodimers formed upon expression of the deletion mutants seemed to be lower for basically all mutants, and also to vary between individual constructs. In particular, the already mentioned constructs like pS6-pS11 and pS12a and pS12b showed reduced homodimerization efficiency. However, since the detected amount of monomer was also considerably reduced for several constructs, instability of the deletion mutants has to be considered. It is therefore difficult to thoroughly quantify the results. Importantly, the analysis shows that within the scale of the deletions tested here, no region within the E^rns^ sequence was identified to be crucial for homodimerization. Thus, none of the 14 residue deletions covered an essential interaction motif, so the observed transient interaction of E^rns^ monomers obviously employs larger parts of their sequence.

### 3.3. Dimerization Occurs with Cys Residues Located at Different Positions

**Figure 3:**
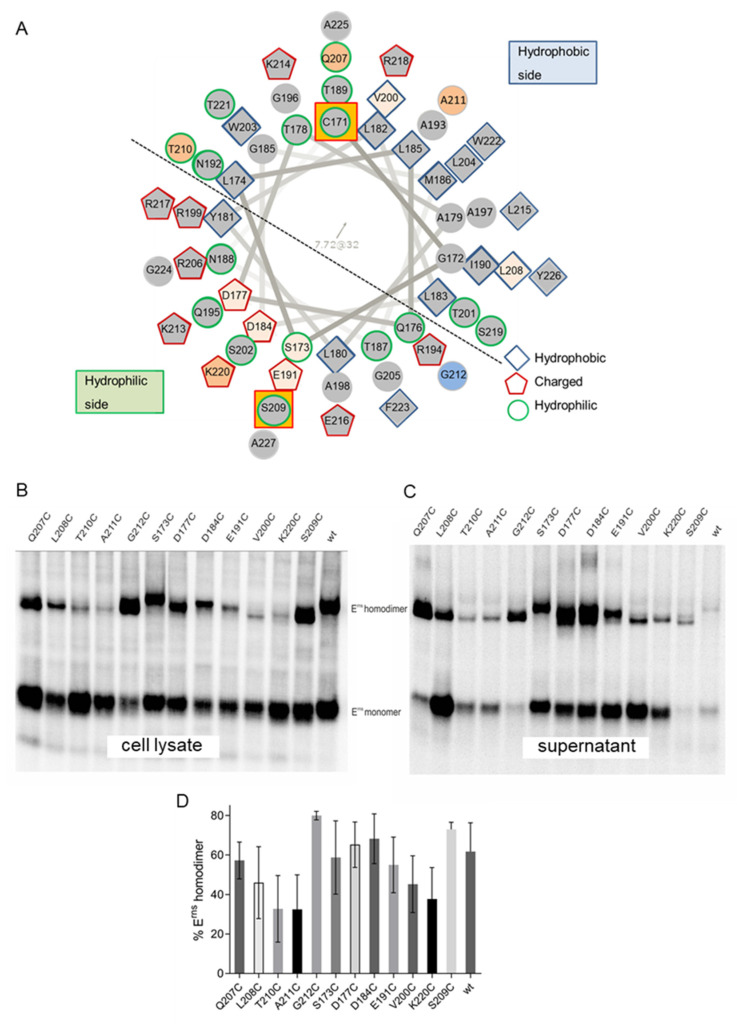
Disulfide bond formation can occur between various Cys residues introduced into the E^rns^ membrane anchor region. (**A**) Helical wheel representation of the E^rns^ membrane anchor region from amino acid C171 to the carboxy-terminal A227 (original scheme established using software http://rzlab.ucr.edu/scripts/wheel/wheel.cgi (accessed April 2015), subsequently edited by hand). Hydrophobic, uncharged hydrophilic and charged amino acids are highlighted as indicated. A dotted line indicates the location of the hydrophobic and hydrophilic sides of the helix as deduced from biochemical character of the amino acids and the results of NMR analyses [27] (the amphipathic character of this helix is not ideal, so that some polar residues are found on the hydrophobic side and vice versa). The key residues C171 and S209 are highlighted by an orange square. Residues mutated in experiments presented in sections (**B**–**D**) are indicated by a colored instead of grey background of the symbol, with darker orange for positions at which mutation leads to decreased dimerization, and a blue background for position 212 showing enhanced dimer formation. Please note that both the amino-terminal and carboxy-terminal regions of the sequence included in this representation extend beyond the core region, for which α-helical folding was proven [27], so that these parts might not be helical. (**B**) Results of an immunoprecipitation experiment of proteins from extracts of cells transiently expressing E^rns^ with the indicated mutations. Precipitation was done with E^rns^-specific monoclonal antibody 24/16. Precipitates were treated with PNGase F prior to separation by 10% SDS-PAGE under nonreducing conditions. (**C**) Equivalent to (B), but conducted with supernatant of the transiently expressing cells. (**D**) Quantification of the E^rns^ dimer/monomer ratio determined for the indicated mutants. Quantification was done via phosphorimager analysis of in situ radioactively-labelled proteins immunoprecipitated from cell extract and supernatant. Total values determined for E^rns^ monomer and dimer in supernatant plus total values for cell extract gave 100% expression product from which the percentage of dimer (supernatant plus cell extract) was calculated. The graph represents the results of at least three independent expression experiments. Standard deviation is shown. Results of statistic evaluation are described in the main text.

An interesting finding of previous work was initiated by identification of pseudorevertants in infected animals, which contained an S209C exchange together with the original C171Δ or C171F mutations. E^rns^ with this pseudoreversion again formed homodimers. Viruses with the respective mutation partially regained virulence proving that this exchange was phenotypically relevant [41]. From a molecular point of view, this finding was interesting since the Cys at position 209 is located on the hydrophilic side within the center of the amphipathic helix that binds in plane to the membrane surface and thereby hooks the protein to the lipid bilayer [27,29,30] (Figure 3A). Thus, the formation of a disulfide bond at this position is somewhat surprising. Since pseudoreversion was only observed at position 209, we speculated whether this position is privileged for structural reasons for disulfide bond formation, or whether the somewhat similar biochemical property of serine and cysteine was the reason for detection of this change in several animals. We therefore established E^rns^ mutants with a deletion of C171 and replacement of residues around position 209 by Cys. Upon transient expression, immunoprecipitation and nonreducing SDS-PAGE, we identified homodimers for all of these mutants (Figure 3B). However, in the analyzed cell extracts the monomer/dimer ratio was obviously different for the mutated proteins when compared to the wt protein or the mutant protein with Cys at position 209. In a previous report we showed that exchange of amino acids in the E^rns^ membrane anchor can dramatically increase secretion of the protein [10], which could bias the results. We therefore also looked for the secreted proteins and found that, in some cases, the reduced level of intracellular homodimer was compensated by a higher amount of secreted dimer, but for others comparison of the percentage of total amount of dimer was significantly lower than for wt or mutant C171Δ/S209C (Figure 3B,C). In particular, a comparison of the results obtained for mutants with Cys introduced at positions around residue 209 with the data for the original mutant recovered from the animals revealed a statistically significant impairment of homodimerization for Cys at positions 207, 210, and 211 (*p* values 0.04, 0.01 and 0.01, respectively; darker orange background in Figure 3A), whereas the mutant G212C formed homodimers more efficiently than the S209C variant (*p* value 0.02; blue background in Figure 3A) (Figure 3D). Looking again at the helical wheel model of the E^rns^ membrane anchor (Figure 3A), it becomes obvious that positions 207, 208 and 211 are located at the hydrophobic side of the amphipathic helix, which is embedded within the lipid bilayer, whereas 210 and 212 are found close to the hydrophobic/hydrophilic border. These results indicate that the position of a Cys around the axis of the amphipathic helix influences the homodimerization rate, but a clear pattern for enhanced or reduced dimer formation is not obvious. Importantly, all mutants with a Cys in the sequence around position 209 were able to establish homodimers quite efficiently despite different positions with regard to the helix axis.

As a next step, we made equivalent changes at positions quite distant from 209 to check whether the region around position 209 is preferred for dimer formation for some unknown reason. We chose residues upstream (177, 184, 191, 200) or downstream (220) for this approach. Again, all mutants were able to establish disulfide-linked homodimers (Figure 3B,C). Since some of the exchanges affected residues known to be important for intracellular retention of E^rns^ [10], the secretion levels of these mutants were generally high (Figure 3C). This was also the case for those residues not tested with regard to their role in retention so far (S173, V200 and K220). However, when the percentage of total amount of homodimer was calculated, the tested changes at positions 173, 184, 191 and 200 were not considerably hampered in dimerization (Figure 3D). Only exchange of K220, a position located at the carboxy-terminal border of the determined helical part of the E^rns^ membrane anchor, significantly reduced dimerization (compared to the wt protein *p* value 0.04; compared to the mutant C171Δ/S209C *p* value 0.01). Taken together, homodimer formation via disulfide bonds is possible between Cys residues at a large range of positions within the amphipathic helix and flanking regions with only rather low variation in efficiency.

### 3.4. Disulfide Bond Formation between C171 and C209 Is Not Detected

**Figure 4:**
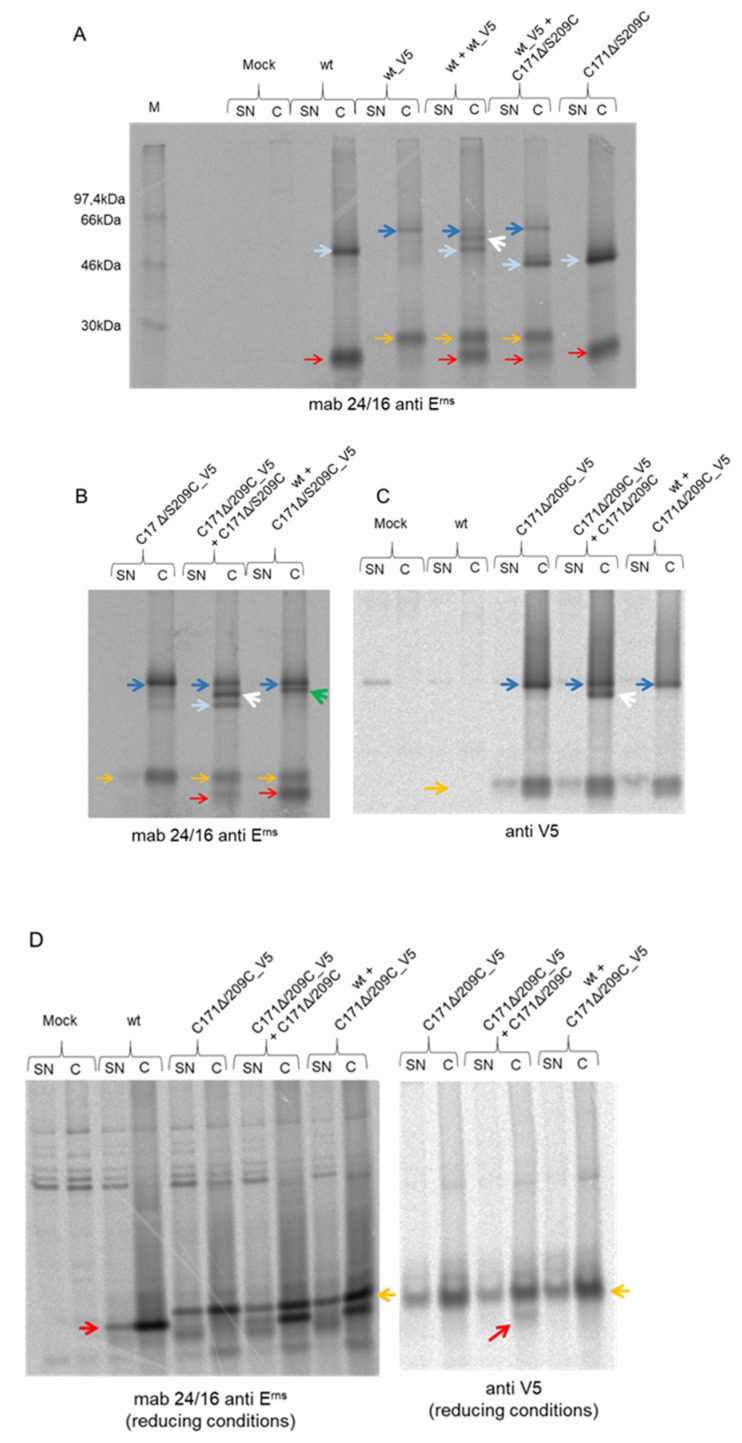
Co-immunoprecipitation experiments of E^rns^ wt and mutants with or without carboxyterminal V5-tag. (**A**) wt E^rns^ and wt E^rns^ with V5-tag (wt_V5) were expressed separately or together with each other. In addition, wt_V5 was expressed with E^rns^ mutant C171Δ/S209C. The proteins were labeled in situ, precipitated with mab 24/16 against E^rns^, and analyzed by nonreducing SDS-PAGE. Red arrows: E^rns^ monomers; orange arrows: E^rns^_V5 monomers; light blue arrows: E^rns^ dimers; dark blue arrows: E^rns^_V5 dimers; white arrow: heterodimers of E^rns^ and E^rns^_V5. Please note that the homodimer composed of two mutant proteins moves considerably faster on the gel than the wt-wt homodimer (**B**) Equivalent to (**A**), but with mutant C171Δ/S209C_V5 instead of wt_V5. The green arrow points at a band running close to the C171Δ/S209C_V5 homodimer that does not comigrate with any of the control bands. (**C**) Same cell extracts/supernatants as in (**B**), but precipitated with an anti-V5 serum. The band marked with a green arrow in (**B**) is not detected here. (**D**) Equivalent to (**C**), but with SDS-PAGE conducted under reducing conditions. In addition to extracts of cells transiently expressing the desired proteins, we also analyzed the supernatant of these cells since E^rns^ is known to be secreted to a certain extent into the cell free supernatant. However, this analysis did not yield results influencing the conclusions of the experiments and are therefore not discussed in detail.

The apparently high degree of flexibility with regard to positions allowing efficient disulfide bond formation between the membrane anchor regions of two E^rns^ monomers prompted us to test whether Cys residues located at different positions in significant spatial distance were also able to establish a S-S bridge. To be able to differentiate between the two different monomers expressed simultaneously in one cell, we cloned a construct displaying the wt sequence followed by a stretch coding for a V5-tag (wt_V5) as already described for BVDV E^rns^ before [40]. To prevent cleavage of the tag by signal peptidase we introduced a mutation at the codon in the minus three position at the 3′end of the E^rns^ gene. When wt_V5 was expressed alone, both monomer and dimer were detected. The migration rate of both forms was considerably reduced compared to the wt protein bands without the tag (Figure 4A). When the V5-tagged wt protein was expressed together with the wt protein lacking the tag, the expected pattern was observed with three dimer bands [rom top: wt_V5-wt_V5 (dark blue arrow), wt-wt_V5 (white arrow) and wt-wt (light blue arrow), Figure 4A] and two monomers of different size. The combination of wt_V5 and C171Δ/S209C yielded only two prominent dimer bands, one comigrating with wt_V5-wt_V5, the other comigrating with C171Δ/S209C-C171Δ/S209C. The latter band showed a considerably enhanced migration rate in comparison with the wt-wt homodimer, whereas the monomer bands comigrated. This finding points at a significant change of the dimer protein structure due to the disulfide at position 209 resulting in enhanced electrophoretic mobility. The C171Δ/S209C-C171Δ/S209C band was accompanied by a weak band almost comigrating with the main band which might be an aberrant product. The composition of this band cannot be deduced, but it is obvious that it migrates too fast for the wt_V5-C171Δ/S209C dimer (see also below). Similarly, a construct C171Δ/S209C_V5 was established and co-expressed with different partners. The combination of C171Δ/S209C_V5 and C171Δ/S209C resulted again in detection of three dimer bands representing the possible combinations of the two partner proteins (Figure 4B). C171Δ/S209C_V5 co-expressed with wt E^rns^ yielded only two bands, representing the dimers composed of two mutant proteins and the wt-wt dimer (the latter marked with a green arrow in Figure 4B). For further analysis, we repeated the precipitation with a commercial anti-V5-tag serum (Figure 4C) using the same cell extracts as before in Figure 4B. On the resulting gel, the combination of C171Δ/S209C and C171Δ/S209C_V5 showed only two dimer bands as expected (dimers of two proteins with V5-tag and the combination of a monomer with and a second monomer without the V5-tag, whereas the dimer without V5 cannot be recognized by this serum) and one monomer band. The combined expression of wt and C171Δ/S209C_V5 resulted in detection of only one dimer band. The equivalent results were obtained for the reciprocal experiment with the tagged wt and the mutant protein without tag corresponding to Figure 4A (not shown). Thus, the band marked with a green arrow in Figure 4B indeed represents the wt-wt homodimer. Taken together, the results clearly show that a dimer composed of one monomer with C171 and the second monomer with C209 is not generated.

We wanted to make sure that the bands identified in Figure 4A–C indeed represented dimers covalently linked via disulfide bonds and, therefore, separated some of the precipitated products also under reducing conditions. Precipitates generated with the E^rns^ specific mab 24/16 showed the expected banding pattern in cell extracts with a faster migrating band in all samples derived from cells expressing proteins without V5 and a band of higher molecular weight in all cases where a V5 fusion protein had been expressed (Figure 4D). The analysis of supernatants of transfected cells showed the same pattern together with an additional band of unknown origin. When the V5 antibody was used for precipitation, two bands were only visible for the combination C171Δ/S209C and C171Δ/S209C_V5 (Figure 4D), demonstrating that the faster running dimer identified for this combination in Figure 4C was indeed composed of these two proteins covalently linked via an S-S bridge. As expected, the band representing the protein without the tag was much weaker here, since the smaller protein can only be co-precipitated, whereas the tagged version is precipitated, regardless whether it is present as part of the two different dimers or as monomer. Taken together, the results of these experiments proved that disulfide-linked dimers could only be formed between E^rns^ monomers containing the Cys residue at either position 171 or 209.

### 3.5. Formation of Disulfide Bonds between Closely Located Cys Residues

**Figure 5:**
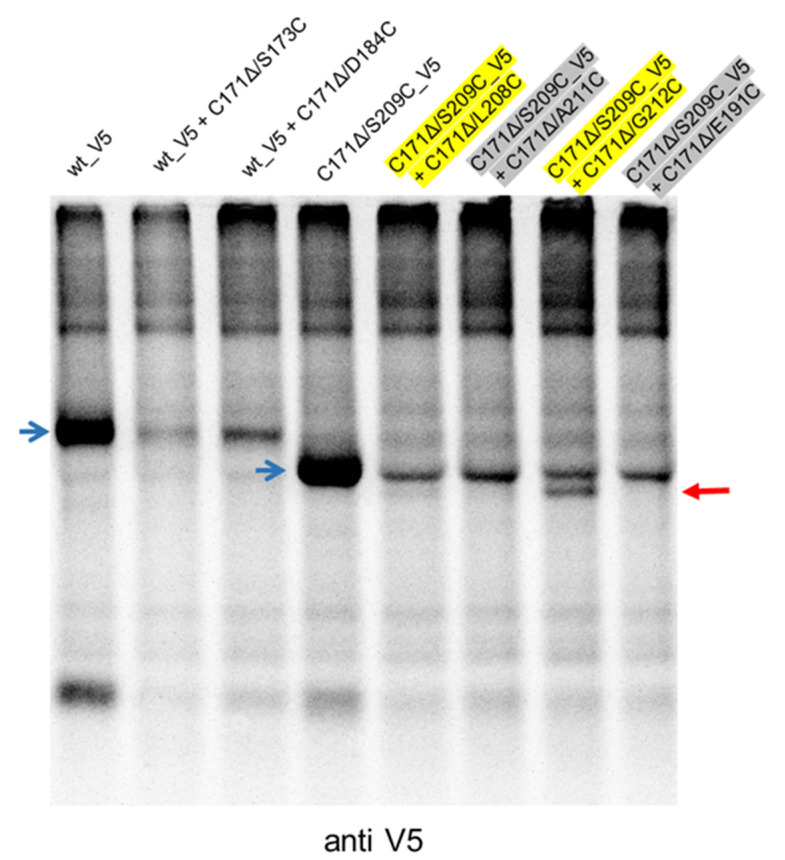
Co-immunoprecipitation experiments of E^rns^ mutants with Cys at different positions. E^rns^ wt_V5 was transiently expressed in cells either alone (utmost left lane) or together with the indicated two E^rns^ mutants containing a deletion of C171 in combination with Cys at position 173 or 184 (second and third lane, respectively). Similarly, the five lanes on the right show the results for E^rns^ mutant C171Δ/S209C_V5 alone or in combination with the indicated mutants. For better readability, the complex names of the constructs shown in the right four lanes were highlighted in different colors. Precipitation was done with the anti-V5 serum. Samples were separated via 10% SDS-PAGE under nonreducing conditions. Blue arrows point at E^rns^ homodimers of two monomers containing V5. The red arrow on the right side of the gel labels a heterodimer most likely composed of one monomer with a V5 tag and one monomer without a tag. Because of the complex construct names in the right 4 lanes, yellow and grey shadows are used to make it easier to identify which construct is shown in which lane.

The absence of disulfide bond formation between C171 and C209 shown in the above-described experiments could be due to the long distance between the two residues or structural constraints. To test whether Cys residues located at different but closer positions could form a covalent binding, we expressed wt_V5 together with mutants C171Δ/S173C or C171Δ/D184C. In addition, we also tested the combinations of C171Δ/S209C_V5 with C171Δ/L208C, C171Δ/A211C, C171Δ/G212C or C171Δ/E191C. Precipitation with the anti-V5-tag serum resulted in detection of homodimer bands composed of two molecules E^rns^ containing a V5-tag, since all these bands comigrated with the band detected when the respective V5-tagged protein was expressed alone (Figure 5). The homodimers wt_V5-wt_V5 and C171Δ/S209C_V5-C171Δ/S209C_V5 showed significantly different migration rates, which should again be due to differences in 3D structure as already discussed for the versions without the tag in context of Figure 4. Only for the combination C171Δ/S209C_V5 with C171Δ/G212C was a second, faster moving dimer band observed in addition to the homodimer of two tagged monomers. The latter mutant had shown already highly efficient homodimerization when expressed alone (Figure 3). Accordingly, position 212 might be a preferred site for disulfide bond formation in general. However, a more logical explanation would be that the two crucial residues at position 209 and 212 are in rather close vicinity and both point towards the watery surrounding (see also Discussion). 

## 4. Discussion

The E^rns^ protein of pestiviruses represents one of the most fascinating viral proteins with its combination of functions as an essential structural protein and a secreted virulence factor [6,11]. We have shown that the latter function depends on the intrinsic RNase activity of this protein [18,25,35]. It has long been proposed that the secretion of E^rns^ from the infected cells is crucial for its virulence factor function. This hypothesis was based on the facts that E^rns^ is an envelope protein already translocated to the ER lumen during translation, and that formation of the enzymatically active RNase depends on the post-translational modifications like disulfide bond formation and glycosylation occurring only upon translocation [6]. However, a formal proof for the dependency of the E^rns^ virulence factor activity on its secretion is still missing. 

Another early observation was the formation of disulfide-linked E^rns^ homodimers within infected or transfected cells [7,37]. Biochemical and crystal structure analyses revealed that only one Cys, namely the residue at position 171, was not engaged in intramolecular disulfide bonds [38,52]. C171 is the only Cys that is not absolutely conserved among pestiviruses, even though the number of virus isolates lacking this Cys is very low [40]. Mutation analysis with pestiviruses that normally contain C171 revealed that both CSFV and BVDV can be propagated when lacking this residue, but CSFV lacking C171 was attenuated in the natural host, indicating a connection between E^rns^ homodimerization and pestivirus virulence [40]. This hypothesis was supported by the finding that pseudorevertants which regained the ability to form E^rns^ homodimers by a S209C mutation also partially regained virulence [41]. The function of homodimerization of E^rns^ with regard to induction of clinical signs is still not clear. It might be that this effect is due to a higher resistance of the dimeric RNase to inhibition via host-derived RNase inhibitors, as reported for the bovine seminal RNase [53,54,55,56].

The recovery of viable engineered virus mutants lacking C171 raised the question of whether monomers of these altered proteins did not interact at all or formed noncovalently stabilized homodimers that were either transient or not stable enough to be detected. Our crosslink experiments show that transient interaction of two monomers obviously occurs, since we were able to specifically crosslink E^rns^ monomers to dimers. In contrast to HCV that expresses non-covalent E1/E2 heterodimers stable enough to be detected on denaturing SDS-PAGE [42,46,47,57,58,59], the non-covalent E^rns^ homodimers were only detectable after crosslinking. Neither gel electrophoresis nor co-immunoprecipitation analysis demonstrated the existence of dimers in the absence of Cys in the membrane anchor region. It, therefore, can be concluded that E^rns^ forms only transient and unstable homodimers when covalent linkage is missing.

Nevertheless, the above-described experiments revealed that E^rns^ monomers should make contacts transiently. As we show here, this is not due to a locally defined contact region but seems to involve at least larger areas of the protein, since consecutive deletions of 14 amino acids did not lead to detection of dimerization negative mutants, so that a crucial localized contact area affected by the loss of a significant number of residues seems not to exist. Unfortunately, the crystal structure analysis of E^rns^ amino acids 1–165 did not provide data on the homodimer, so its structure and the contact sites between the monomers are still obscure [38].

The identification of a dimerization positive pseudorevertant with Cys at position 209 [41] raised some important questions with regard to structural and biochemical constraints. First of all, it was not clear whether 209 is the only alternative position suited for disulfide bond formation or whether the exchange Cys for Ser was preferred because of the biochemical similarity of these two amino acids. To answer this question, we tested a variety of synthetic mutants with Cys residues introduced at different positions of the E^rns^ carboxy-terminus. Surprisingly, all tested variants were able to build homodimers and the overall efficiency was not dramatically different from what was determined for the wt protein.

A more general and difficult to answer question concerns the structural arrangement that allows the formation of a disulfide bond between two residues located within an α-helix that exhibits restricted flexibility with regard to rotation around the helix axis due to interaction of the hydrophobic side of its amphipathic helix with the lipid bilayer. Our data show that, in contrast to position 209, residue 171 is situated outside of the helical part of the E^rns^ membrane anchor region [27]. It can be hypothesized that the region around C171 is exposed to the hydrophilic surrounding and is somewhat flexible to rotate and bend. Thus, dimers formed via C171 can be imagined to have their membrane anchor helices in various orientations to each other from extended in opposite directions to parallel alignment. According to our NMR analyses [27], R194 and R199 are considered to frame the hydrophilic side of the helix. In this setting, residue 209 is located in the central part of the hydrophilic surface (see Figure 3A). It can be hypothesized that close alignment of the two helices and partial rotation around the helix axis are necessary to allow disulfide bond formation between two residues at position 209. The 3D structure of this unusual product seems to be clearly different from the original wt homodimer, since the former moves on the gel much faster than the latter. Disulfide bond formation between Cys residues introduced at other positions can be expected to encounter the same problem, which in the case of residues located on the hydrophobic side of the amphipathic helix is even severer since the respective residues should be immersed in the lipid phase. We were not able to deduce a pattern of correlating efficiency of dimer formation with the position of the individual mutated residues in the helix. Thus, the general conclusion that disulfide bonds can probably be established with Cys residues introduced at all positions within the E^rns^ membrane anchor region seems more important than the possible meaning of some variation in efficiency of dimer formation.

With one exception, all mutants analyzed here were able to establish dimers only with a second monomer of the same type. In principle, two amphipathic helices bound in plane to the surface of a membrane could align in parallel or antiparallel to bring individual residues in intermolecular contact. Both arrangements could allow contact between two residues located at different helix positions by shifting one helix with respect to the other. However, this seems not to occur for most of the combinations, so that one can conclude that the exact position within the helix is crucial for disulfide bond formation. This might be explained in a way that the interaction between the two helices is somehow strictly fixed. Since in such a fixed interaction two residues located at the same but variable position of their helix can only get in contact when the helices align in parallel, this scenario clearly supports a parallel arrangement with exactly matched amino- and carboxy-termini of both helices.

The only exception of the rule described above was detected for a combination of mutant C171Δ/S209C with mutant C171Δ/G212C. This combination represents the only one for which both Cys residues are located in close vicinity with regard to their position along the helix axis and on nearly the same side of the helix (see helical wheel representation in Figure 3A). Thus, the distance between these two residues in both longitudinal and cyclic position might represent the “swing” that is allowed in the parallel alignment of two E^rns^ monomers.

Taken together, our results show that there are no structural constraints preventing disulfide bond formation between Cys residues at different positions of the E^rns^ membrane anchor region as long as these cysteines are located at the same position of the sequence or a very short distance away. We have shown recently that mutation of a variety of amino acids (especially charged residues) in the membrane anchor region of E^rns^ prevents the recovery of infectious viruses, and that at least one defect resulting from these tested exchanges concerns the assembly and budding step of pestiviruses [10,60]. Another recent publication reported that exchanges of positively charged residues in the anchor region can block virus entry and also transport of secreted E^rns^ into target cells [61]. With this knowledge in mind, it is rather likely that isolation of S209C as the only pseudorevertant is not necessarily a consequence of structural aspects but could simply be due to impairment of functions of the E^rns^ membrane anchor by other mutations leading to non-viable viruses. It might well be, however, that the strict alignment of the long amphipathic helices of E^rns^ monomers plays a crucial role for membrane binding and bending, important for different steps in the viral live cycle and its interaction with cells of the immune system of the host. Future work with appropriately designed virus mutants is necessary to get more insight into the biological aspects of this fascinating system.

## Data Availability

All data are presented in the manuscript.
